# Overview on the Mycotoxins Incidence in Serbia in the Period 2004–2016

**DOI:** 10.3390/toxins10070279

**Published:** 2018-07-05

**Authors:** Bozidar Udovicki, Kris Audenaert, Sarah De Saeger, Andreja Rajkovic

**Affiliations:** 1 Faculty of Agriculture, Department of Food Safety and Food Quality Management, University of Belgrade, Nemanjina 6, 11080 Zemun-Belgrade, Serbia; bozidar.udovicki@agrif.bg.ac.rs; 2 Faculty of Bioscience Engineering, Department of Plants and Crops, Ghent University Campus Schoonmeersen, Valentin Vaerwyckweg 1, B-9000 Ghent, Belgium; kris.audenaert@ugent.be; 3 Faculty of Pharmaceutical Sciences, Department of Bioanalysis, Laboratory of Food Analysis, Ghent University, Ottergemsesteenweg 460, B-9000 Ghent, Belgium; Sarah.DeSaeger@UGent.be; 4 Faculty of Bioscience Engineering, Department of Food Technology, Food Safety and Health, Ghent University, Coupure links 653, B-9000 Ghent, Belgium

**Keywords:** cereals, dairy products, incidence, mycotoxins, risk assessment, climate, risk assessment

## Abstract

With an average annual production of 6.9 M tonnes and 2.5 M tonnes of maize and wheat respectively, Serbia is one of the main grain producers and exporters in Europe. Cereals are also the major staple food in Serbian diet. In view of the high cereal consumption, for human and animal nutrition, the presence of mycotoxins entails a high public health risk of chronic exposure to mycotoxins. This study provides an overview of the incidence of predominant mycotoxins, mainly in cereal and dairy products, in Serbia, in the 2004–2016, using data reported in the scientific literature. The study demonstrated that the total prevalence of aflatoxins was 62.9% (*n* = 12,517) with 26.2% of the samples exceeding the EU limits during this period. Results obtained for T-2/HT-2 (*n* = 523), deoxynivalenol (*n* = 2907), fumonisins (*n* = 998), zearalenone (*n* = 689) and ochratoxin A (*n* = 740) indicated the prevalence of 45.5%, 42.9%, 63.3%, 39.3% and 28.1%, respectively. For these mycotoxins, the EU limits were less frequently exceeded. Comprehensive collection and analysis of all accessible information reviewed in this paper showed moderate incidence and prevalence of mycotoxins in Serbia, with an exception of the 2012 drought year and the 2014 flood year.

## 1. Introduction

Fungi produce a large number of secondary metabolites, such as plant growth regulators, pharmaceutically useful compounds, pigments and mycotoxins, which do not always have an obvious biological function [1] but may represent a certain ecological advantage for the fungi. It is known that agricultural commodities and particularly cereals are prone to fungal infection during the growth of the crop or during harvest, transport or storage. As a result, agricultural commodities are often contaminated with mycotoxins leading to acute and chronic health exposure. Such exposure may result in acute visible symptoms but can also result in long-term hidden health damages. Mycotoxins are mainly produced by the species of *Aspergillus*, *Penicillium, Alternaria, Claviceps* and *Fusarium* [2]. They are rather stable under most food processing conditions and their complete elimination from the contaminated material is fairly difficult. A first step in reducing the exposure to mycotoxins through food and feed are baseline studies that provide an overall image of mycotoxin incidence. Not only contamination of cereals used for food is of great concern but also mycotoxin contamination of feed must be taken into account. The possible carry-over of each mycotoxin raises the concern of safety of food of animal origin and potentially contributes to mycotoxin intake in humans [3]. Many surveys have been carried out to evaluate the incidence of mycotoxin contamination worldwide. Food and Agriculture Organization of the United Nations estimated that approximately 25% of the world’s cereals are contaminated with mycotoxins [4]. On a global level, 30% to 100% of food and feed samples are contaminated [5,6,7,8,9]. These reported percentages could be also an underestimation if modified mycotoxins are taken into consideration. These modified mycotoxins can result from partial metabolisation of mycotoxins by plants or can originate during food and feed processing [10,11]. In addition, very little is known about the actual contribution of modified forms to the total number of mycotoxins found in cereals and products thereof. Therefore, occurrence data are needed to calculate exposure and to assess the risk posed by total mycotoxins in cereals. For both modified and unmodified mycotoxins the impact of food processing and household handlings needs to be quantified to establish real intake data and at the time same in vitro and in vivo toxicity and toxicokinetics of (modified) mycotoxins are needed [12,13].

Maize and wheat are the most widespread crops in Serbia. With an average annual production of 6.9 million tonnes and 2.5 million tonnes for maize and wheat, respectively (averaged data for period 2014–2016), Serbia is one of the prominent grain producers and exporters in Europe, primarily due to maize exports. An average export is estimated at 2.2 million tonnes of maize and 0.6 million tonnes of wheat per year [14], with exceptional years such as 2016 with an export of almost 1 million tonnes of wheat. For the same period import of both maize and wheat was negligible [15,16]. Moreover, cereals production, especially of wheat and maize, is an important social and economic activity and accounts for 68% of all land used for agricultural purposes [17]. Cereals are consumed through a variety of foods, with the largest fraction belonging to bread consumption [18]. Wheat, primarily intended for human consumption, is the dominant crop in many regions of the country and is the most important staple food in Serbia. It accounts for approximately 17% of the total sown surface [14]. Total domestic consumption (human consumption, seeds and feed) of wheat in Serbia is estimated to be approximately 1.5 million tonnes annually with wheat for human consumption estimated at 1.2 million tonnes annually and per capita consumption at 180 kg, which is significantly higher than consumption levels in most European countries [16]. Maize accounts for approximately 30% of the total sown surface [14] and represents the most important component of feed. Serbia usually consumes approximately 4.5 million tonnes of maize annually, with the vast majority being used for animal feed (4.2 million tonnes) and 200,000–300,000 tonnes used for human consumption [16]. While export consignments are subjected to inherently stringent phyto-sanitary controls on the borders, one should with the same level of diligence and scrutiny verify the safety of cereals sold on local markets. Stringent mycotoxin standards on exported foods mean that some exporting nations are likely to export their best-quality raw materials while keeping contaminated foods domestically, which advertently results in higher risk of mycotoxin exposure of local population [19]. Our recent data on Malawian groundnut value chain showed that such hypothesis is not irrelevant as local market products may contain distinctly higher mycotoxin levels than export lots [20].

Serbia is located in the moderate continental climate belt, where the most frequently isolated fungi contaminating cereals, vegetables and fruits belong to *Fusarium*, *Penicillium* and *Aspergillus* genera. Since mycotoxin contamination of food and feed raw materials is hardly avoidable, occurrence data of mycotoxins is of great importance for food safety. However, the availability of data concerning the distribution of mycotoxins in cereals and cereal-based products produced in Serbia is scattered and limited. Moreover, most of the data seem to originate from the Northern part of Serbia, Vojvodina, the largest wheat and maize producer in the country (in terms of surface and the yield), creating biased information on the overall spread of fungal and mycotoxin pressure in agricultural commodities. While climate and changes thereof are not under an immediate farmers’ control, agronomic factors, such as the type of hybrid, tillage, crop rotation, as well as postharvest management (type of storage and storage handling) are factors influencing fungal growth and toxinogenesis and they are under farmers’ control. Therefore, coherent prevalence data, appropriate training of farmers and food business operators and application of degradation and detoxification techniques would be very valuable in the risk management of mycotoxins [21,22].

While national legislation of Serbia on mycotoxins is harmonised with European legislation [23], a small number of conducted systematic monitoring programs provide insufficient data about the occurrence of mycotoxins in Serbia and therefore in-depth analysis of exposure and legal compliances cannot be provided. Therefore, the main aim of this study is to provide an estimation of the presence of principal mycotoxins in food and feed in Serbia using different data sources. This is the first comprehensive review of the mycotoxins incidence in Serbia since the work of Levic et al. [24] who participated in drafting a general report on toxigenic fungi and mycotoxins in Europe [25].

## 2. Principal Mycotoxins in Food and Feed in Serbia

### 2.1. Aflatoxins

Aflatoxins (AFs) B1, B2, G1 and G2 are four naturally occurring AFs produced by various strains of *Aspergillus*, mainly by *Aspergillus flavus*, *A. parasiticus*, *A. nomius* and *A. tamarii* [2]. AFs are furanocoumarins with immunotoxic, mutagenic and carcinogenic effects and the toxicity is mainly caused by the lactone ring and the difuran ring [26]. Aflatoxin B1 (AFB1) is the most carcinogenic and best-studied aflatoxin. Aflatoxin M1 (AFM1) is the 4-hydroxy derivative of AFB1, formed in the liver and excreted in the milk and the mammary glands of both humans and lactating animals that have been fed with AFB1 contaminated diet [27].

Considering that Serbia has a moderate continental type of climate, there has been a general understanding that only a low risk of AFs contamination is present. Occurrence data for *Aspergillus* spp. in Serbia show low incidence frequency and low levels in grain in previous years. Over the period 1967–2008 the frequency varied from 1.0 to 23.1% [28]. This probably provides a rationale on why only a few studies of AFs occurrence were conducted in Serbia prior to 2012. Furthermore, those studies have shown no or a low amount of AFs in various food and feed commodities [29,30,31,32,33,34,35,36,37,38]. However, in 2012 Serbia had prolonged drought during spring and summer which might have contributed to the high contamination frequency and concentration of AFs in maize and consequently in milk and dairy products. In February–March 2013 several European countries, including Serbia, Croatia and Romania reported nationwide contamination of milk for human consumption (and possibly of derived products) with AFs. It was reported in March of the same year that feeds originating from Serbia and imported in The Netherlands, Belgium and Germany were contaminated. As a result of the reduced yield and AFs contamination, Serbian maize export plummeted significantly in comparison to previous and subsequent years [15,39]. During March 2013, the maximum permissible limit (MRL) for AFM1 was temporally and without transparent risk assessment, changed from 0.05 μg kg^−1^ to 0.5 μg kg^−1^ in order to help struggling dairy industry. In following years MRL was changed several times and currently is set at 0.25 μg kg^−1^ [40]. In 2013, Serbian authorities sent 48 samples of milk to the European Union Reference Laboratory for Mycotoxins in RIKILT-Wageningen (The Netherlands) and results showed that 33 samples exceeded EU MRL for AFM1 in milk. In conclusion of the report, the results were described as alarming. This large-scale incident inspired many research efforts, official controls and self-monitoring of the dairy producers in the subsequent period.

Based on published data analysis, the total prevalence of AFs in the period 2007–2016 was 62.9% in 12,517 samples tested with 26.2% of the samples exceeding the EU MRL. In the period from 2007 to 2011 prevalence of AFs was 15.6% in 628 samples of various commodities with only 1.3% of the samples exceeding the EU MRL corresponding to seven raw goat milk samples and one barley flakes sample [34,38]. Most of the positive samples were in the year 2012 and in the subsequent years. In this period, the prevalence of AFs in all types of analysed samples was 65.4% (*n* = 11,889) with 27.5% of the samples exceeding the EU MRLs (full data not shown). Among the products analysed in 2012 and in prior years, AFs were most frequently found in maize and maize-based products. Maize and maize-based products samples analysed prior to 2012 had an AFs prevalence of 9.7% (*n* = 351) with none of the samples exceeding the EU MRL [30,31,32,36,37,38]. The samples analysed in 2012 had an AFs prevalence of 49.9% (*n* = 724), with 33% of the samples exceeding the EU MRL and with the mean values ranging from 18.15 µg kg^–1^ to 36.3 µg kg^−1^ [37,41,42,43,44].

Analysis of publications made in following years indicated that a primary screening focus was on milk and dairy products. On one hand these products are an important part of average Serbian diet and on the other hand they are a key vector for intake of AFM1. The total prevalence of AFM1 in this period was 67.8% (*n* = 10,781), with 27.6% of the samples exceeding the EU MRL. Increased official monitoring of farms and milk collecting stations in 2013 (data not included in overall results) revealed the average AFM1 prevalence in raw milk of 45.7% (*n* = 2045) [44]. More precise details of post-2012 AFs occurrence in Serbia including relative mean values of concentration, concentrations range and reviewed data lots are presented in Table 1.

The overall prevalence of AFs in Serbian cereals for the investigated period was considerably higher than the global prevalence reported by Andrade & Caldas [55]. Based on the review from these authors the global AFs prevalence in cereals for the 2000−2015 period was 37.6% (*n* = 18,097), with maize accounting for 54.3% of the samples. The mean aflatoxin level found in positive samples, considering all cereals, was 34.2 ± 3.4 µg kg^−1^. Additional analysis by the same authors was performed using GEMS/Food database which had shown the occurrence of 12.7% (*n* = 4536), with a mean of 10.7 ± 35.3 µg kg^−1^. Taking into consideration only maize contamination in Europe, AFs prevalence was 23.4% (*n* = 1858) [55], comparing to 36.8% (*n* = 1075) reported in Serbia for the 2007–2012 period. However, the best comparison can be made when comparing results from neighbouring countries with similar climate and within the similar period investigated. Total AFB1 prevalence in maize and maize-based feed for the 2009–2013 period in Croatia was 31.4% (*n* = 972) with 21.7% of samples exceeding EU MRL [56]. In the maize samples from 2012, AFB1 was detected in 38.1% (*n* = 633) of samples, with 28.8% of the samples containing AFB1 at levels higher than the EU MRL. The AFs prevalence determined in both countries within the entire period are comparable, while results from 2012 results showed the noticeable higher prevalence of both positive samples and samples that were above EU MRL in Serbia, showing 49.9% (*n* = 724) and 33%, respectively. Prevalence of positive milk samples collected in Croatia from February to July 2013 was 46.1% (*n* = 3716) and 39.5% (*n* = 706), with 27.8% and 9.64% of samples exceeding EU MRL for raw and UHT milk respectively [57]. Study for the period February 2013–January 2014 from Macedonia had shown the prevalence of AFM1 in raw milk of 42.4% (*n* = 3636) with only 2.9% exceeding EU MRL [58]. In the same period, the prevalence of AFM1 positive milk samples from Serbia was 87% (*n* = 1036), with 60.8% of the samples above EU MRLs. However, as the number of analysed samples was not the same and no information is available on sampling protocols direct comparisons should be cautiously interpreted.

### 2.2. Fusarium Mycotoxins

*Fusarium* species require lower temperatures for growth and mycotoxins production than the AFs producing *Aspergillus* species and mycotoxins from *Fusarium* species have traditionally been associated with temperate climate regions. They are predominantly associated with Fusarium Head Blight in wheat and other cereals all over Europe [59]. *Fusarium* species synthesise a wide range of mycotoxins of diverse structure and chemistry, comprising trichothecenes (T-2 toxin, HT-2 toxin, diacetoxyscirpenol (DAS), deoxynivalenol (DON), DON-3-glucoside (DON-3-Glc), 15- and 3-acetyl-deoxynivalenol (ADONs), nivalenol (NIV) and fusarenon-X (FUS-X)), zearalenone (ZEN) and fumonisins (FUMs) [60].

*Fusarium* spp. are frequently found in the Serbian climatic area which is suitable for cereal production [61]. Agri-climatic conditions in Serbian farming, including among others weather conditions at flowering, preceding crop, no or minimal tillage, susceptible cultivar and use of last year grains as a seeding material influence occurrence of *Fusarium* spp. and related mycotoxins [62]. The prevalence of the genus *Fusarium* has been determined on wheat grain in Serbia but the composition and the intensity of occurrence of certain species have been varying over the years [63]. *Fusarium graminearum* or *F. culmorum* (both producing DON and *F. graminearum* producing ZEN) were found on wheat grain in various periods since the 1960s. *F. graminearum* was encountered each year with variable intensity. This was not the case with *F. culmorum* [24]. *F. graminearum* growth and DON production is recognised worldwide during cool and wet summers [64] *F. verticillioides* and FUMs are found more frequently in years with weather conditions not favoring the growth of other *Fusarium* species, for example, higher temperatures [24]. The greatest outbreaks of epidemic melds, mostly caused by *Fusarium* species, were registered in maize in 1955, 1968, 1972, 1974 and 1984 and in wheat in the early 1970s [65]. The greatest outbreaks of animal diseases, especially diseases of pigs such as estrogenism, vomiting and feed refusal, dermal toxicity and others were recorded during the same and/or subsequent years [66]. The large-scale diseases of animals were not recorded after the late 1980s, apart from some problems in animal feeding in certain farms in northern Serbian province caused by very high levels of ZEN [24]. Since the first survey of DON occurrence in Serbian crops from 2004/05 production years [65], numerous studies on the natural occurrence of Fusarium mycotoxins in food were conducted and published providing valuable insight into the occurrence of main Fusarium mycotoxins in Serbia.

Thanks to many efforts done to analyse established Fusarium mycotoxins in Serbian cereals future studies are made possible. In years to come efforts should be made to analyse also emerging Fusarium mycotoxins beauvericin and enniatins, which prevalence we have previously reported in wheat and maize originating from Poland and Hungary (as well as Nigeria and Zimbabwe) [67]. Also, other authors have confirmed the relevance of these emerging mycotoxins in Central and Eastern Europe [68]. These authors reported that enniatin B presented the highest incidence with 41% in wheat and 32% in wheat-based products and maximum levels of 815 μg kg^−1^ and 170 μg kg^−1^ in wheat and wheat-based products, respectively.

#### 2.2.1. Trichothecenes

Trichothecenes are a large family of chemically related mycotoxins produced by various species of *Fusarium*, *Myrothecium*, *Trichoderma*, *Trichothecium*, *Cephalosporium*, *Verticimonosporium* and *Stachybotrys*. All mycotoxins in this group have small molecular weights, the same basic ring structure and a characteristic 12,13-epoxide group [69]. The trichothecenes are subdivided into four basic groups (A, B, C, D), with types A and B representing the most important members. Type A trichothecenes do not contain a carbonyl (keto) function at C8 (T-2 toxin, HT-2 toxin, DAS). Type B trichothecenes have a carbonyl (keto) group at C8 (DON, DON-3Glc, ADONs, NIV and FUS-x).

T-2/HT-2 toxins are predominantly produced by *F. sporotrichioides* and *F. langsethiae* [70]. As T-2 toxin is readily metabolised to HT-2 toxin these two mycotoxins are frequently co-occurring. In the period 2005–2016, the total prevalence of T-2/HT2 was 45.5% (*n* = 523), with 10.9% of the samples exceeding the EU MRL. T-2/HT2 occurrence data in Serbia including relative mean values of T-2/HT2 concentration, concentrations range and reviewed data lots are presented in Table 2.

The overall prevalence of T-2/HT-2 toxins in Serbian cereals for the investigated period was slightly higher compared to the prevalence of these mycotoxins in samples collected between 2005 and 2010 from 22 European countries [77]. A total of 17,683 analytical results for the T-2 toxin, 16,536 for HT-2 toxin and 20,519 for the sum of T-2 and HT-2 were collected, with an overall 65% of results below limit of detection (LOD) or limit of quantification (LOQ). The highest mean concentrations for the sum of T-2 and HT-2 toxins in food, feed and unprocessed grains were observed in grains and grain milling products. Comparing results from unprocessed wheat grains of the undefined end-use category, from which the large proportion of Serbian samples were comprised, T-2 and HT-2 were found in 24% (*n* = 4799) and 30% (*n* = 4471) of the samples, respectively. Reported mean levels were in the range of 1.7–8.0 (lower/upper bound) and 3.7–8.8 μg kg^−1^ (lower/upper bound), for T-2 and HT-2 respectively.

DAS, produced by *F. sporotrichioides* and *F. poae*, is less toxic then T-2/HT2 but it can cause adverse effects in farm animals [78]. Only one research was conducted on the presence of DAS in Serbia [59]. It was found in 9 out of 31 samples of crop wheat (29%), in the range of 31.0–125.0 μg kg^−1^ and with a mean value for positive samples of 61.0 μg kg^−1^ and the median value of a 62.0 μg kg^−1^.

DON is produced most commonly by *F. graminearum* and *F. culmorum* [59]. Although DON is among the least toxic of the trichothecenes, it is the predominant trichothecene throughout the world and its occurrence is considered to be an indicator of the possible presence of other, more toxic trichothecenes [79].

In the period 2004–2016, the total prevalence of DON in Serbian cereals was 42.9% (*n* = 2907), with 11.3% of the samples exceeding the EU MRL. DON was reported present, in variable concentrations, in every year since 2004 with a low number of samples exceeding EU MRL. An exception was the year 2014 and, to some extent, the year 2010. The results obtained by Kos et al. [80] and Jajic et al. [81,82] indicated that weather conditions recorded in 2014 and 2010, in terms of air temperature and the amount of precipitation, had a significant influence on DON occurrence. Samples analysed in 2014 showed a DON prevalence of 96.2% (*n* = 640), with 45.6% of the samples exceeding the EU MRL (Table 3), while samples analysed in 2010 showed a DON prevalence of 73.5% (*n* = 310), with 6.8% of the samples exceeding the EU MRL (Table 3). Apart from these years, the DON prevalence in samples analysed during the investigated period was 20.5% (*n* = 1957) with only 0.8% of the samples exceeding the EU MRL. DON occurrence data in Serbia including relative mean values of DON concentration, concentrations range and reviewed data lots are presented in Table 3.

In a report composed by EFSA [88], a total of 18,884 samples collected by 21 Member States and Norway between 2007 and 2012 were evaluated. DON was found in 44.6%, 43.5% and 75.2% of unprocessed grains of undefined end-use, food and feed samples, respectively. It was most frequently quantified and at the highest levels in maize, wheat and oat grains and derived food and feed products, compared to the other varieties of cereals. The level of DON exceeded EU MRL in 0.8% of the food samples and guidance values in 1.7% of the feed samples. Considering category most analysed in Serbia (unprocessed grains of undefined end-use) overall prevalence of DON was in a similar range. DON was found in around half of the samples of barley, maize, oats, rye and wheat analysed (*n* = 975). It was less frequently quantified in other cereals (buckwheat, millet, rice, spelt). The highest levels were found in maize and wheat, with average middle bound levels slightly higher than 300 μg kg^−1^, followed by oat and barley, with average middle bound levels around 150 μg kg^−1^.

DON-3-Glc a modified derivative and plant phase II metabolite of DON occurs in naturally contaminated wheat, maize, oat and barley. A major concern is the possible hydrolysis of the DON-3-Glc conjugate back to its toxic precursor DON during mammalian digestion [89]. Only one research was conducted on the presence of DON-3-Glc in Serbia [72]. It was found in 7 out of 54 samples of crop wheat (12.9%), in the range of 17.0-83.0 μg kg^−1^ and with a mean value of 5.0 μg kg^−1^ (for samples that were below LOD half of LOD value was taken in calculating the average value) and with median value bellow LOD. Samples analysed in this research were negative on the presence of NIV, ADONs and FUS-X.

#### 2.2.2. Fumonisins

FUMs (FB1, FB2 and FB3) are structurally similar to sphingolipid long-chain bases such as sphinganine and sphingosine. This feature is tightly related to their toxicity mechanism through the inhibition of the sphingolipid biosynthesis [26]. The unsubstituted primary amino group at C2 competitively inhibits ceramide synthase, thereby disrupting the biosynthesis of ceramide and sphingolipid metabolism [90]. Fumonisin B1 (FB1) is the most prevalent member of this group mainly produced by *F. verticillioides* and *F. proliferatum* [2].

In the period 2005–2016, the total prevalence of FUMs (total FUMs or FB1) was 63.3% (*n* = 998), with only 0.2% of the samples exceeding the EU MRL. FUMs prevalence in wheat and maize after harvest during the investigated period was 65.9% (*n* = 126) and 88.5% (*n* = 148) respectively. FUMs prevalence was higher in stored wheat having an incidence of 71.0% (*n* = 283) while in stored maize prevalence was lower amounting to 72.6% (*n* = 215). FUMs occurrence data in Serbia including relative mean values of FUMs concentration, concentrations range and reviewed data lots are presented in Table 4.

The overall prevalence of FUMs in Serbia, for the investigated period, was slightly higher compared to the prevalence of FUMs in samples collected between 2000 and 2010 and reported by 11 European countries [92]. Prevalence of FUMs in grains and grain-based products was 47% (*n* = 2981) and in grain milling products was 53% (*n* = 1366), with a reported mean level in the range of 170–215 μg kg^−1^ (lower/upper bound) and 279–315 μg kg^−1^ (lower/upper bound), respectively.

#### 2.2.3. Zearalenone

A common feature of many *Fusarium* species is their ability to synthesize ZEN. It is primarily produced by *F. graminearum* and *F. culmorum* [2]. ZEN is classified as an estrogenic mycotoxin. The toxicity of ZEN is mainly conferred by its lactone group and the free C-4 hydroxyl group which is necessary for binding the oestrogen receptor [93].

In the period 2005–2016, the total prevalence of ZEN was 39.3% (*n* = 689), with 9.7% of the samples exceeding the EU MRL. ZEN prevalence in wheat and maize after harvest during the investigated period was 46.3% (*n* = 201) and 7.3% (*n* = 124) respectively, with 20.4% and 0% of the samples exceeding EU MRL. ZEN prevalence was higher in both stored wheat (*n* = 103) and maize (*n* = 40) having an incidence of 90.3% and 55% respectively, with 6.8% and 0% of the samples exceeding the EU MRL. ZEN prevalence in other crops, grains, processed maize and wheat-based food and feed materials was 24.4% (*n* = 221) with 8.6% of the samples exceeding EU MRL (mostly in maize-based food or feed). ZEN occurrence data in Serbia including relative mean values of ZEN concentrations, concentrations range and reviewed data lots are presented in Table 5.

The overall prevalence of ZEN in Serbia for the investigated period was considerably higher than the prevalence reported in Europe in a similar period. A total of 13,075 analytical results obtained on food samples and 9877 analytical results obtained on unprocessed grains samples, sampled by 19 European countries in 2005–2010, shown prevalence of ZEN of 15% [95]. The highest concentrations of zearalenone were reported for wheat bran, corn and products thereof. In the category of unprocessed grains of unknown end-use ZEN was found in 38% (*n* = 5318) and 56% (*n* = 2460) of wheat and maize samples, respectively. Reported mean levels have been in the range of 22–27 μg kg^−1^ (lower/upper bound) and 76–87 μg kg^−1^ (lower/upper bound) for wheat and maize, respectively.

### 2.3. Ochratoxins

Ochratoxins are a group of mycotoxins sharing an isocoumarin moiety substituted with a phenylalanine group (OTA, OTB and hydroxyl-OTA), a phenylalanine ester group (OTC, OTA methyl ester, OTB methyl ester and OTB ethyl ester) or a hydroxyl group (OTα and OTβ) [26]. Ochratoxin A (OTA) is the most significant member of the group because of its incidence in food and feed. It is composed of a 7-carboxy-5-chloro-8-hydroxy-3, 4-dihydro-3-R-methylisocoumarin (OTα) moiety and the amino acid L-phenylalanine group with both structures linked through a carboxy group via an amide bond [26]. OTA is produced by *Aspergillus* and *Penicillium* species mainly by *Penicillium verrucosum*, *A. ochraceus* and *A. carbonarius* [2]. OTA in food and feed is sometimes accompanied by the non-chlorinated analogue, ochratoxin B which is much less toxic. 

Data on the prevalence of OTA producing species in Serbia are scarce and limited to a small number of studies [84,96]. Next, to the cereals and other insufficiently dried commodities, OTA has been detected in pig’s blood, kidney, liver muscle and adipose tissue with rather high levels found in animals suffering from porcine nephropathy, especially in countries of the Balkan Peninsula [33]. Nephropathy in pigs with characteristic macroscopic changes of the type “mottled or pale enlarged kidneys” has been frequently identified at meat inspection in Serbia, corresponding well with the data found in other countries of the Balkan Peninsula [33].

In the period 2008–2016, the total prevalence of OTA was 28.1% (*n* = 740), with 3.0% of the samples exceeding the EU MRL. The screening of the presence of OTA was mainly focused on commodities which can be used directly as food or feed. Reported OTA prevalence in food commodities was 24.5% (*n* = 629), with 2.1% of the samples exceeding EU MRL. The OTA prevalence in feed commodities was 49.5% (*n* = 111), with 8.1% of the samples exceeding EU MRL. The occurrence data for OTA in Serbia including relative mean values of OTA concentrations, concentrations range and reviewed data lots are presented in Table 6.

OTA presence in Serbia was consistent during a period of years at considerable levels and in various food and feedstuffs. Still, published data are insufficient in order to make a comparison between OTA prevalence in Serbia and other countries.

### 2.4. Mycotoxins Co-Occurrence

Effects of individual mycotoxins on animal and human health are well known and documented, however, there is a rising concern from possible multiple mycotoxins contamination and toxicological effects thereof. As most fungi are able to simultaneously produce a number of mycotoxins and commodities can be contaminated by several fungi, the co-occurrence of mycotoxins is likely to occur, thus, humans and animals are generally not exposed to one mycotoxin but to several toxins at the same time [97]. The toxicity of mycotoxins combinations cannot always be predicted from their individual toxicities. The original data on combined toxic effects of mycotoxins are limited and therefore the health risk from this multi-exposure is not well-known [97]. Risk assessment of combined exposure to multiple chemicals is defined as a priority objective by European Food Safety Authority (EFSA) and EU [98]. Among the 116 reported possible mycotoxins combinations found by the different authors in cereals and derived cereal product samples, AFs + FUMs, DON + ZEN, AFs + OTA and FUMs + ZEN were the most present ones [99]. Among the combinations, the AFB1 + FB1 mixture has received the greatest attention over the last decade as they are two of the most relevant mycotoxins co-occurring in maize with most severe adverse health effects.

Although the occurrence of multiple mycotoxins was observed in several studies, very few studies actually were focused on co-occurrence of mycotoxins. Kos et al. [74] reported a 53.3% and 2.2% incidence (*n* = 90) of T-2/HT-2 and FUMs and T-2/HT-2, FUMs and DON co-occurrence in maize samples, respectively. In the study on mycotoxins in stored maize grains [30] co-occurrence of AFB1, FB1, DON and ZEN was found in 100% of the samples (*n* = 12). Krnjaja, et al. [83] reported a 10% incidence (*n* = 20) of DON and ZEN co-occurrence in winter wheat samples. Stankovic et al. [71] reported incidence of a co-occurrence of FB1 with DON, T-2 and ZEN in 78.6%, 60.7% and 67.9%, respectively, in wheat samples collected in 2005 (*n* = 28) and 86.7%, 52.0% and 88.0%, respectively, in wheat samples collected in 2007 (*n* = 75). Another research by Stankovic et al. [59] on wheat samples collected in 2005 (*n* = 31) showed the incidence of a co-occurrence of ZEN + DAS + T-2, ZEN + DAS, ZEN + T-2 and DAS + T-2 in 6.5%, 3.2%, 9.7% and 16.1% of the samples respectively. Jaksic et al. [61] reported the incidence of a co-occurrence of DON and FUMs in 36% of wheat samples (*n* = 75) and in 91.7% of maize samples (*n* = 24). The incidence of a co-occurrence of Fusarium mycotoxins determined in 54 winter wheat samples analysed by Skrbic et al. [72] was 12.96% for DON + DON-3-Glc and 3.7% for DON + HT-2.

### 2.5. RASFF Notifications

The EU has one of the highest food safety standards in the world—largely thanks to risk assessment based food safety legislation in place and preventive farm-to-fork approach. Among key tools to achieve this is Rapid Alert System for Food and Feed (RASFF). Created in 1979, RASFF enables information to be shared efficiently between its members (EU-28 national food safety authorities, Commission, EFSA, ESA, Norway, Liechtenstein, Iceland and Switzerland) and provides around-the-clock service to ensure that urgent notifications are sent, received and responded to collectively and efficiently. Analysis of the RASFF database provides useful trend analyses such as identification of transgressor and detector nations and determination of seasonal variations in contamination patterns.

Out of 22 mycotoxins related alerts via the RASFF system (period 2004–2016) concerning food and feed of Serbian origin, only 16 were concerning food directly produced in Serbia. A partial reason for this relatively low number, considering recent mycotoxins outbreaks, amount of grain exported and period investigated, could be in the fact that large proportion of Serbian wheat and maize were not exported in countries covered by RASFF system notifications in terms of notifying countries.

## 3. Adverse Effects and Exposure Assessment

### 3.1. Aflatoxins

In humans, the risks associated with AFs consumption are well documented and the International Agency for Research on Cancer (IARC) has designated AFB1 as a human liver group 1 carcinogen and AFM1 as a group 2B carcinogen [100]. The risk of liver cancer in individuals exposed to chronic hepatitis B virus infection and aflatoxin is up to 30 times greater than the risk in individuals exposed to aflatoxin only [101]. The Joint FAO/WHO Expert Committee on Food Additives (JECFA) did not specify Tolerable Daily Intake (TDI) for AFs; they concluded that daily exposure even with a concentration lower than 1 ng kg^−1^ bw, contributed to the risk of liver cancer [102]. The potency of AFM1 is ten folds lower than the potency of AFB1 [102] and in common use is TDI of 0.2 ng kg^−1^ bw per day proposed by Kuiper-Goodman [103].

In Serbia, there is lack of consumption data for maize and other commodities that are prone to contamination with AFB1. Furthermore, data on the AFB1 presence in these commodities are limited to a small number of studies and for many of them, the distribution of concentrations is not reported. These two aspects comprise the main components of exposure assessment. Skrbic et al. [45] estimated intakes of AFM1 for the Serbian adults as 1.420, 0.769 and 0.503 ng kg^−1^ bw per day during February, April and May of 2013, respectively, through consumed milk. Similar results were obtained by Kos et al. [46] showing average intake for the adult population in the range of 0.49–0.56 ng kg^−1^ bw per day in 2013. Milicevic et al. [51] calculated average intake in 2015–2016 for the adult population in the range of 0.18–0.366 ng kg^−1^ bw per day for females and 0.197–0.402 ng kg^−1^ bw per day for males calculated based on AFM1 content in heat treated and raw milk, respectively. Both of two later studies have shown highest intake of AFM1 and therefore health risk, for the category of infants (1–4 years old) due to their higher intake level of milk and lower body weight. However, this data did not fully take into account differences in the consumption patterns of different consumer groups, or the variability in the concentration of aflatoxins across different product categories and therefore does not represent probabilistic exposure assessments. Nevertheless, the findings showed a higher exposure for the Serbian adult population in 2013 in comparison with the estimate of AFM1 intake from milk in the European regional diet of 0.11 ng kg^−1^ bw per day [102] and TDI value proposed by Kuiper-Goodman.

### 3.2. Fusarium Mycotoxins

IARC classified T-2/HT2 toxins as group 3 carcinogens [100]. They are known to impair protein and DNA synthesis and to induce haematotoxicity and myelotoxicity associated with impairment of haematopoiesis in bone marrow [77]. Other toxic effects include dermal toxicity, developmental and reproductive toxicity and neurotoxicity [77]. T-2 toxin is the principal causal toxin in the human alimentary toxic aleukia [104]. The estimated total chronic dietary exposures to the sum of T-2 and HT-2 toxins across 14 European countries for adults was in the range from 0.0034 to 0.018 μg kg^−1^ bw per day for average consumers. In the elderly population, the chronic dietary exposure was slightly lower compared to other adults. The highest chronic dietary exposure estimates are for toddlers at 0.012 to 0.043 μg kg^–1^ bw per day for average consumers. Grains and grain-based foods, in particular, bread, fine bakery wares, grain milling products and breakfast cereals, made the largest contribution [77]. Estimates of chronic dietary exposure for populations of all age groups to the sum of T-2 and HT-2 toxins based on the available occurrence data are below TDI of 0.1 μg kg^−1^ bw per day.

IARC classified DON as group 3 carcinogen [100]. The main effects of long-term dietary exposure of animals to DON are weight gain suppression, anorexia and altered nutritional efficiency [88]. Depending on the population group, chronic dietary exposure of children to DON (upper bound) was estimated to be on average between 0.54 and 1.02 μg kg^−1^ bw per day and at the 95th percentile between 0.95 and 1.86 μg kg^−1^ bw per day. Chronic dietary exposure of adolescents, adults, elderly and very elderly to DON (upper bound) was estimated to be on average between 0.22 and 0.58 μg kg^−1^ bw per day and at the 95th percentile between 0.43 and 1.08 μg kg^−1^ bw per day depending on the population group [88]. In almost all population groups, the main contributor to the total chronic exposure was bread and roll followed by pasta and fine bakery wares. The exposure assessments conducted at national or European level concluded that high consumers and young children were exposed to DON at levels close to or even higher than the TDI of 1 μg kg^−1^ bw per day [88].

IARC classified FUMs as group 2B carcinogens [100]. There are no confirmed records of acute FB1 toxicity in humans but it has been linked to oesophageal cancer [105] and liver cancer [106]. The exposure to FUMs across European countries could be of concern, especially in children age groups. At high exposure, the maximal exceedance was 2.5-to 3-fold the Provisional Maximum Tolerable Daily Intake of 2 μg kg^−1^ bw per day [92].

ZEN has been implicated as a causative agent in epidemics of premature breast development in girls in Puerto Rico and south-eastern Hungary [107,108]. Taking into consideration that ZEN is produced by *Fusarium* species that usually also produce other mycotoxins, co-occurrence with other Fusarium mycotoxins, particularly DON and FUMs is regularly observed and it raises important issues regarding additivity and/or synergism in the aetiology of mycotoxicosis in animals and humans. IARC classified ZEN as group 3 carcinogen [100]. Estimates of chronic dietary exposure to zearalenone based on the available occurrence data were below or in the region of the TDI (0.25 μg kg^−1^ bw) for all age groups and not a health concern [95]. The estimated chronic total dietary exposures to ZEN of adults across 19 European countries, using lower bound and upper bound concentrations, ranged from 0.0024 to 0.029 μg kg^−1^ bw per day for average consumers. The highest chronic exposure was estimated in toddlers ranging from 0.0093 to 0.1 μg kg^−1^ bw per day for average consumers [95].

Currently, there are no exposure assessment studies concerning Fusarium mycotoxins in Serbia. Furthermore, data on consumption are limited to reports on wheat consumptions per capita [16]. In order to obtain exposure assessment to Fusarium mycotoxins for (different segments of) Serbian population more informative monitoring studies must be made. For this purpose, occurrence and concentrations data in various food groups at ready to eat form are needed. The analysis in raw materials must be treated with due care to avoid biased overestimations that do not take the impact of milling and processing [88]. Furthermore, in order to fully assess exposure to Fusarium mycotoxins data consumption for various wheat products and different population groups must be included in the analysis. This approach should result in reported ranges of average dietary intakes (possibly as a percentage of the TDI values) for different subpopulations of consumers.

Taking into consideration data on prevalence and mean levels obtained in this review we can only presume higher exposure rates for the population in Serbia, particularly concerning the exposure to DON and FUMs.

### 3.3. Ochratoxins

The IARC classified OTA as group 2B carcinogen [100]. OTA is a potent nephrotoxin and causes both acute and chronic effects in the kidneys of all mammalian species tested, although the mechanism is uncertain. Exposure to OTA has been associated with distinct renal diseases endemic in the Balkans, referred to as Balkan Endemic Nephropathy and Urinary Tract Tumours [109]. Despite these facts, there were an insufficient number of studies and published reports on OTA presence in food and feed in Serbia. JEFCA concluded in 2008 that the epidemiological and clinical data available do not provide a basis for calculating the likely carcinogenic potency in human and that Balkan Endemic Nephropathy may involve other nephrotoxic agents [110]. The current average dietary exposure levels to OTA in Europe have been determined by JECFA to be 8–17 ng kg^−1^ bw per week based on processed cereals, compared with 25 ng kg^−1^ bw per week in the previous evaluation, based on raw cereals, well below the proposed Tolerable Weekly Intake [110].

## 4. Control Strategies

Quantitative information presented in the current study shows the presence of mycotoxins in the wide range of cereal commodities, including those from primary production and products for direct human consumption. Many of the reported findings indicate poor implementation or even absence of control strategies, both in the prevention of on-field contamination and intervention in the post-harvest phases. Furthermore, the incidental appearance of *A. flavus* in maize during 2012 in Serbia demonstrated the weakness of the control system, as well as the weakness of the (implementation of) national legislation [111].

In general, main control strategies that need to be developed and implemented should include structured monitoring of mycotoxins presence, prevention of fungal and mycotoxin contamination and decontamination of mycotoxins present in food and feed. Several reviews have been published in recent years providing insights into the latest developments in pre-harvest and post-harvest mycotoxin management options for different commodities [69,112]. Moreover, over twenty years European Commission has funded more than 200 research projects that were targeting fungal and mycotoxin contamination providing some state-of-the art approaches to manage mycotoxins across the food chain.

In Europe, food safety regulatory systems are effective and efficient at identifying contaminated materials and removing them from the primary food chain. European Commission set strict regulation for maximum levels for mycotoxins and methods for sampling and analysis for the official control of the mycotoxins levels [113,114]. Detection of mycotoxins is mostly performed by conventional chromatographic-based and immunological-based methods, usually requiring extensive sample preparation procedures and trained personnel. Recently, microfluidic “lab-on-a-chip” devices have been developed and they have a great potential for accurate and high-throughput detection of mycotoxins in agricultural and food products [115]. The effectiveness of monitoring is most evident through significant improvement of milk and dairy products safety in 2015 and 2016 compared to previous years indicating that dairy processors responded to the pressure aroused from the crisis in 2013 and took more responsibility by refusing the reception of contaminated raw milk from farmers [52].

Several codes of practice have been developed by Codex Alimentarius [116] for the prevention and reduction of mycotoxins in cereals, peanuts, apple products and raw materials, based on Good Agricultural Practice and Good Manufacturing Practice. Some of the recommendations for the reduction of mycotoxins in cereals through several stages of production include: crop rotation, destroying of debris, use of fertilizer and/or soil conditioners, seeding fungi resisting varieties, scheduled crop planting, weeds control (at planting stage); containers should be clean and dry, determination of moisture content, when applicable drying of the crop (at harvest); avoiding piling of wet, freshly harvested crops, dry and well-vented protective structures, fast drying, mycotoxins monitoring during storage, air circulation, documented procedures (storage).

Various physical and chemical strategies have also been developed to help prevent mycotoxin contamination, including physical separation, fluorescence sorting, extraction with sorbents and adsorption [112]. While a range of chemical compounds, including hydrochloric acid, ammonia, hydrogen peroxide, O3, sodium bisulphite and chlorine seem to hold great potential in the detoxification of mycotoxins unfortunately their use significantly decreases the nutritional value of the foods or produces toxic derivatives in the treated product with undesirable sensory properties severely limit their widespread use [112]. Recently, there has been an increasing interest in the use of bacteria, yeast and fungi to help reduce the toxic effect of mycotoxins.

In the recent years, there has been increased interest and effort in creating predictive models as an additional tool for control of mycotoxins occurrence. Climate change has been reported as a driver for emerging food and feeds safety issues worldwide and its expected impact on the presence of mycotoxins in food and feed is of great concern [117]. In the perspective of a dramatic switch of the toxigenic fungal population profiles, as recorded by several scientists over a wide range of geographical areas and in various types of crops, a modelling approach could become a powerful tool for a more efficient management of mycotoxin contamination worldwide [117].

## 5. Conclusions

Comprehensive collection and analysis of all accessible information reviewed in this paper showed moderate incidence and prevalence of mycotoxins in food and feed in Serbia, with an exception of the 2012 drought year and the 2014 flood year. The number of samples that were above limits set by EU legislation was relatively low. Still, in most cases, prevalence and/levels for most of the mycotoxins reviewed here are above the European standards. With an exception of AFs in recent years, a number of analysed samples for over a 10 year period is relatively low, biasing a reliable estimate of mycotoxins prevalence and concentrations in certain food and feed commodities. At the moment, insufficient data hampers the possibility of in-depth risk assessment study for multifaceted risk management. First steps towards a consolidated Mycotoxins Risk Profile in Serbian diet are well planned and regular baseline surveys in state-wide surveillance programs. The resulting data on incidence and mycotoxin levels must be coupled with climatic and agro-technical metadata, including spatial and temporal dimension. Statistical assessment of confounding factors is required. Second step are national food consumption databases using EFSA models. The third step is counteracting exposure bias by introducing a correct dilution/concentration factor of typical milling and industrial/household processing instead of using initial concentration in raw cereals. Finally, the risk rankings for different mycotoxin/commodities/target consumer groups and quantitative risk assessment are needed to support new risk management strategies. For aggregative and cumulative exposure, all routes and commodities need to be taken into account in national strategy for mycotoxin management but the optimization of resources needed, will come from previously determined risk rankings and/or quantitative risk assessment studies. Next to established mycotoxins, care must be given to the current lack of information on emerging mycotoxins. Furthermore, the toxicological hazards of each mycotoxin are to be assessed from the literature and Serbian epidemiological data for a correct estimate of weight of evidence as to the mycotoxin being attributable to human disease. The study of tissue distribution, bioaccumulation, carry-over, persistence, transference of mycotoxins, as well as, toxicokinetics and ADME (absorption, distribution, metabolism and excretion), through direct assessment of mycotoxins or mycotoxin markers, are essential for accurate risk assessment and in particular to establish the oral dose (intake from food), the internal dose (biologically active) and the dose-response relationship and therefore regular base-line surveys are needed across different crops, geographic and climatic areas, as well as food consumption surveys. Especially care needs to be taken to collect data that will allow assessments of aggregative and cumulative exposures for mycotoxins with similar mode of action. Additionally, nationwide risk management strategies need to be implemented. The later will ideally rely on prevention and minimization of pre-harvest and post-harvest mycotoxin contamination of food and feed raw materials. The training and education, as well as control and incentive measures, along the cereal value chain, particularly among the farmers, are the most obvious risk management tools.

## Figures and Tables

**Table 1:** Overview of Aflatoxins (AFs) occurrence after the year 2012.

Type of Commodity	Np/N (%)	Mean ± SD ^b^ (μg kg^−1^)	Range (μg kg^−1^)	Above EU MRL N (%)	Production Year	Reference
Raw milk	558/647 (86.2)	0.308 ± 0.356 ^a^	0.005–1.440	409	2013	[45,46,47]
Heat treated milk	343/389 (88.2)	0.144 ± 0.114 ^a^	0.005–1.200	221	2013	[45,46,47,48]
Total milk 2013	901/1036 (87.0)			630 (60.8)		
Organic milk	6/6 (100)	0.026 ± 0.018	0.010–0.080	1	2013	[46]
Goat milk	10/10 (100)	0.080 ± 0.090	0.008–0.240	4	2013	[46]
Donkey milk	5/5 (100)	0.020 ± 0.020	0.005–0.035	0	2013	[46]
Breast milk	10/10 (100)	0.010 ± 0.006	0.001–0.022	0	2013	[46]
Infant formula	1/22 (4.5)	0.020 ^a^	0.02	0	2013	[46,48]
Commercial white cheese	10/23 (43.5)	0.110	0.130–0.550	3	2013	[49]
Homemade white cheese	9/21 (42.9)	0.080	0.130–0.220	0	2013	[49]
Hard cheese	10/10 (100)	0.640	0.080–2.230	4	2013	[49]
Total products 2013	61/107 (57.0)			12 (11.2)		
Raw milk	30/79 (38.0)	0.035 ± 0.013	0.005 -> 1.000	9	2014	[47]
Heat treated milk	71/165 (43.0)	0.024 ± 0.012 ^a^	0.005 -> 1.000	6	2014	[47,48]
Total milk 2014	101/244 (41.4)			15 (6.1)		
Milk powder	22/67 (32.8)	0.847 ± 1.948	0.005 -> 1.000	17	2013/14	[47]
Yogurt	42/56 (75.0)	0.081 ± 0.092	0.005 -> 1.000	22	2013/14	[47]
Ice cream	14/21 (66.7)	0.071 ± 0.061	0.005 -> 1.000	11	2013/14	[47]
Infant formula	2/33 (66.7)	0.021 ± 0.002	0.005 -> 1.000	2	2013/14	[47]
White cheese	39/47 (83.0)	0.146 ± 0.170	0.005 -> 1.000	28	2013/14	[47]
Hard cheese	21/27 (77.8)	0.379 ± 0.509	0.005 -> 1.000	16	2013/14	[47]
Other	44/71 (62.0)	0.082 ± 0.121	0.005 -> 1.000	28	2013/14	[47]
Total products 2013/14	184/322 (57.1)			124 (38.5)		
Raw milk	1555/2695 (57.7)	0.060 ± 0.95 ^a^	0.004–0.263	801	2015	[50,51,52]
Heat treated milk	364/468 (77.8)	0.027 ± 0.030	<0.005–0.278	43	2015	[51]
Total milk 2015	1919/3163 (60.6)			844 (28.0)		
Dairy products	236/997 (23.7)	0.019 ± 0.024	0.005 0.320	42	2015	[52]
Crop maize (AFs/AFB1)	103/180 (57.2) 103/180 (57.2)	12.700 ± 17.300	1.300–91.400	38 58	2015	[53]
		11.400 ± 14.500	1.300–88.800			
Various breakfast cereals	6/54 (11.1)	0.100 ± 0.0400	0.060–0.150	0	2015	[54]
Total cereals 2015	109/234 (46.6)			58 (24.8)		
Raw Milk	3094/3646 (84.9)	0.069 ± 0.120	<0.005–1.100	1133	2016	[51]
Heat treated milk	753/765 (98.4)	0.039 ± 0.020	<0.005–0.280	171	2016	[51]
Total milk 2016	3847/4411 (87.2)			1304 (29.6)		
Infant formula	23/349 (6.6)	0.011 ± 0.003	<0.005–0.017	1	2015/16	[40]
Milk powder	25/94 (26.6)	0.018 ± 0.010	<0.005–0.035	0	2015/16	[40]
Dairy drinks	13/58 (22.4)	0.034 ± 0.040	<0.005–0.147	3	2015/16	[40]
Total products 2015/16	61/501 (12.2)			4 (0.8)		
Total milk and products	7310/10,781 (67.8)			2975 (27.6)		
Total	7419/11,015 (67.4)			3033 (27.5)		

N—Number of samples; Np—Number of positive samples. ^a^ Mean values and standard deviation of the same type of product and same production year are pooled together using following formulas: Pooled means = (N1 × M1 + N2 × M2 + Nn × Mn + …)/(N1 + N2 + Nn + …); Pooled SD = {(N1 − 1) × S1 + (N2 − 1) × S2 + (Nn − 1)S3 + …}/(N1 + N2 + Nn + …). ^b^ When reported.

**Table 2:** Overview of a T-2/HT2 occurrence in food and feed in Serbia.

Type of Commodity	Toxin	Np/N (%)	Mean ± SD ^b^ (μg kg^−1^)	Range (μg kg^−1^)	Above EU MRN (%)	Production Year	Reference
Crop wheat	T-2	12/31 (28.7)	45.7	31.0–125.0	1	2005	[59]
Stored wheat	T-2	21/28 (75.0)	171.5	60.0–495.0	12	2005	[71]
Stored wheat	T-2	45/75 (60.0)	86.8	86.0–200.0	30	2007	[71]
Crop wheat	HT-2	3/54 (5.6)	9.0 ^c^	128.0–129.0	3	2007	[72]
Crop wheat	T-2	0/54 (0.0)	-	-	0	2007	[72]
Crop wheat	T-2	37/41 (90.2)	24.2	25.0–135.6	3	2010	[73]
Wheat flour	T-2	4/15 (26.7)	4.1 ^c^	9.8–26.9	0	2011	[35]
Wheat flour	HT-2	0/15 (0.0)	-	-	0	2011	[35]
Crop maize	T-2/HT2	48/90 (53.3)	50.9 ± 42.9	25.0–209.0	5	2012	[74]
Stored maize ^a^	T-2	11/29 (37.9)	113.4 ± 92.7	54.7–374.0	0	2012	[43]
Crop maize	T-2/HT2	26/50 (52.0)	25.3	25.3–185.2	3	2012	[75]
Poultry feed	T-2	31/41 (75.6)	55.3	25.1–426.1	0	2014	[76]
Total		238/523 (45.5)			57 (10.9)		

N—Number of samples; Np—Number of positive samples. ^a^ Traditionally stored maize is used only as a feed. ^b^ When reported. ^c^ For samples that were below LOD half of LOD value was taken in calculating the mean value.

**Table 3:** Overview of a deoxynivalenol (DON) occurrence in food and feed in Serbia.

Type of Commodity	Np/N (%)	Mean ± SD ^c^ (μg kg^−1^)	Range (μg kg^−1^)	Above EU MRL N (%)	Production Year	Reference
Stored wheat	2/4 (50.0)	1235.0 ± 856.0	630.0–1840.0	1	2004	[65]
Stored maize ^a^	5/10 (50.0)	536.0 ± 1076.0	40.0–2460.0	1	2004	[65]
Soybean meal	1/13 (7.7)	110.0	110.0	0	2004	[65]
Sunflower meal	4/9 (44.4)	155.0 ± 126.0	40.0–304.0	0	2004	[65]
Crop wheat	4/12 (33.3)	182.0 ± 171.0	57.0–423.0	0	2005	[65]
Crop maize	29/66 (43.9)	363.0 ± 436.0	40.0–2210.0	1	2005	[65]
Soybean meal	1/11 (9.1)	100.0	100.0	0	2005	[65]
Sunflower meal	5/10 (50.0)	447.0 ± 244.0	114.0–788.0	0	2005	[65]
Barley	1/4 (25.0)	140.0	140.0	0	2005	[65]
Stored wheat	24/28 (85.7)	605.5	52.0–3306.0	Nr	2005	[71]
Crop wheat	12/34 (35.3)	223.0 ± 75.0	90.0–410.0	0	2006	[66]
Crop maize	8/21 (38.1)	426.0 ± 396.0	140.0–1340.0	0	2006	[66]
Stored wheat	70/75 (93.3)	282.8	50.0–1090.0	0	2007	[71]
Crop wheat	15/54 (27.8)	33 ^d^	41–309	0	2007	[72]
Crop wheat	3/9 (33.3)	177.0 ± 33.0	142–208	0	2007	[66]
Crop maize	30/119 (25.2)	58.0 ± 39.0	27.0–172.0	0	2007	[66]
Grain food	3/76 (3.9)	923.3 ± 932.4	380.0–2000-0	1	2009	[32]
Crop wheat	20/20 (100)	490.0	110.0–1200.0	0	2009	[83]
Pig feed	10/18 (55.6)	780.0 ± 850.0	250.0–2500.0	Nr	2009	[84]
Total 2004–2009	247/593 (41.7)			4 (0.7)		
Crop wheat	203/271 (74.9)	806.3 ^b^	50.0–5000.0	18	2010	[61,73,81,82,85]
Crop barley	3/15 (20.0)	190.7 ^b^	118.0–355.0	0	2010	[81,82]
Crop maize	22/24 (91.7)	1263.0	154.0–16,528.0	3	2010	[61]
Total 2010	228/310 (73.5)			21 (6.8)		
Wheat flour	13/15 (86.7)	325.0 ^d^	17.5–976.0	1	2011	[35]
Stored maize ^a^	12/12 (100)	128.2	41.0–226.0	1	2011	[30]
Crop maize	2/90 (2.2)	650.0 ± 70.7	600.0–700.0	0	2012	[74]
Stored maize ^a^	7/28 (25.0)	239.0 ± 250.1	82.0–792.0	0	2012	[43]
Crop wheat	13/19 (68.4)	478.0	69.0–918.0		2013	[86]
Crop maize	15/600 (2.5)	642.3 ± 364.7	261.0–1388.0	0	2013	[80]
Total 2011–2013	62/764 (8.1)			2 (0.3)		
Crop maize	576/600 (96.0)	3063.3 ± 1264.4	264.4–9050.0	292	2014	[80]
Crop wheat	40/40 (100)	762.5	175.0–1440.0	0	2014	[87]
Total 2014	616/640 (96.2)			292 (45.6)		
Crop maize	93/600 (15.5)	921.1 ± 952.7	252.3–6280.0	10	2015	[80]
Total of all samples	1246/2907 (42.9)			329 (11.3)		

N—Number of samples; Np—Number of positive samples; Nr—Not reported. ^a^ Traditionally stored maize is used only as a feed. ^b^ Mean values of the same type of product and same production year are pooled together using following formula: Pooled means = (N1 × M1 + N2 × M2 + Nn × Mn + …)/(N1 + N2 + Nn + …). ^c^ When reported. ^d^ For samples that were below LOD half of LOD value was taken in calculating the mean value.

**Table 4:** Overview of Fumonisins (FUMs) occurrence in food and feed in Serbia.

Type of Commodity	Toxin	Np/N (%)	Mean ± SD ^b^ (μg kg^−1^)	Range (μg kg^−1)^	Above EU MRL N (%)	Production Year	Reference
Stored wheat	FB1	23/28 (82.1)	2079.5	750.0–5400.0	Na	2005	[71]
Stored wheat	FB1	69/75 (92.0)	918.8	750.0–4900.0	Na	2007	[71]
Stored maize ^a^	FB1	144/203 (70.9)	1225.7	750.0–4300.0	Nr	2006–2009	[91]
Stored wheat	FB1	109/180 (60.6)	852.7	750.0–4900.0	Na	2008–2009	[91]
Stored barley	FB1	41/120 (34.2)	768.2	750.0–1225.0	Nr	2008–2009	[91]
Crop maize	FUMs	17/34 (50.0)	352.0 ± 460.0	30.0–1520.0	0	2009	[29]
Grain food	FUMs	11/76 (14.5)	282 ± 246.0	56.4–600.0	0	2009	[32]
Crop wheat	FB1	35/41 (85.4)	882.7	750.0–2465.0	Na	2010	[73]
Crop wheat	FUMs	38/75 (50.7)	241.0	27.0–614.0	Na	2010	[61]
Crop maize	FUMs	24/24 (100)	1084.0	60.0–12,880.0	1	2010	[61]
Crop wheat	FB1	10/10 (100)	6286.0	2715.0–16,488.0	Na	2010	[85]
Cornflakes and grits	FUMs	9/15 (60.0)	Nr	25-0–131.0	0	2010	[38]
Wheat flour	FB1/FB2	0/15 (0.0)	-	-	0	2011	[35]
Stored maize ^a^	FB1	12/12 (100)	1610.8	880.0–2950.0	0	2011	[30]
Crop maize	FUMs	90/90 (100)	1730.0 ± 870.0	520.0–5800.0	1	2012	[74]
Total		632/998 (63.3)			2 (0.2)		

N—Number of samples; Np—Number of positive samples; Nr—Not reported; Na—Not applicable. ^a^ Traditionally stored maize is used only as a feed. ^b^ When reported.

**Table 5:** Overview of Zearalenone (ZEN) occurrence in food and feed in Serbia.

Type of Commodity	Np/N (%)	Mean ± SD ^b^ (μg kg^−1^)	Range (μg kg^−1^)	Above EU MRL N (%)	Production Year	Reference
Crop wheat	20/31 (64.5)	133.4	37.0–331.0	13	2005	[59]
Stored wheat	22/28 (78.6)	19.7	10.0–143.0	1	2005	[71]
Stored wheat	71/75 (94.6)	29.0	16.0–201.0	6	2007	[71]
Crop wheat	0/54 (0.0)	-	-	0	2007	[72]
Maize products	25/64 (39.0)	Nr	25.0 -> 200.0	9	2008	[31]
Wheat flour	5/13 (38.5)	Nr	25.0 -> 75.0	2	2008	[31]
Crop maize	9/34 (26.5)	2.7 ± 0.6	1.8–3.4	0	2009	[29]
Grain food	8/76 (10.5)	38.4 ± 13.2	10.6–60.1	0	2009	[32]
Pig feed	8/18 (44.4)	850.0 ± 1416.0	200.0–5000.0	8	2009	[84]
Crop Wheat	2/20 (10.0)	70.0	60.0–80.0	0	2009	[83]
Crop Wheat	37/41 (90.2)	442.6	10.0–1000.0	11	2010	[73]
Complete cow feed	3/16 (18.8)	226.7	220.0–240.0	0	2010	[36]
Other cow feed	0/19 (0.0)	-	-	0	2010	[36]
Crop wheat	24/45 (53.3)	330.0 ± 283.0	68.0–1079.0	17	2010	[94]
Crop wheat	10/10 (100.0)	299.9	157.1–471.1	Nr	2010	[85]
Wheat flour	5/15 (33.3)	4.6 ^c^	1.9–21.1	0	2011	[35]
Stored maize ^a^	12/12 (100)	71.8	15.4–188.1	0	2011	[30]
Crop maize	0/90 (0.0)	-	-	0	2012	[74]
Stored maize ^a^	10/28 (35.7)	73.3 ± 35.3	25.8–130.0	0	2012	[43]
Total	271/689 (39.3)			67 (9.7)		

N—Number of samples; Np—Number of positive samples; Nr—Not reported. ^a^ Traditionally stored maize is used only as a feed. ^b^ When reported. ^c^ For samples that were below LOD half of LOD value was taken in calculating the mean value.

**Table 6:** Overview of an ochratoxin A occurrence in food and feed in Serbia.

Type of Commodity	Np/N (%)	Mean ± SD ^b^ (μg kg^−1^)	Range (μg kg^−1^)	Above EU MRL N (%)	Production Year	Reference
Ground paprika	9/18 (50.0)	Nr	2.00 -> 4.00	0	2008	[31]
Condiments	16/23 (69.6)	Nr	2.00–10.00	0	2008	[31]
Maize products	5/64 (7.8)	Nr	Nr	0	2008	[31]
Cereal flour	0/13 (0.0)	-	-	0	2008	[31]
Pig liver	24/90 (26.7)	0.63 ± 1.87	0.22–14.50	Na	2008	[33]
Pig kidney	30/90 (33.3)	1.26 ± 5.85	0.17–52.50	Na	2008	[33]
Pig blood	28/90 (31.1)	3.70 ± 23.60	0.22–221.00	Na	2008	[33]
Crop Maize	3/14 (21.4)	1.13 ± 0.11	1.07–1.26	0	2009	[29]
Grain food	15/76 (19.7)	4.84 ± 4.49	2.00–15.90	10	2009	[32]
Complete pig feed	9/18 (50.0)	60.00 ± 70.00	60.00–270.00	9	2009	[84]
Maize silage, dry	5/10 (50.0)	15.20	12.00–16.00	Nr	2010	[36]
Other feed for cows	0/25 (0.0)	-	-	0	2010	[36]
Wheat flour	0/15 (0.0)	-	-	0	2011	[35]
Stored maize ^a^	11/28 (39.3)	8.05 ± 2.50	5.03–11.99	0	2012	[43]
Breakfast cereals	17/82 (20.7)	1.76 ± 3.53	0.07–11.81	3	2012	[54]
Chickens feed	16/16 (100)	34.40	19.04–51.30	0	2014	[96]
Hens feed	14/14 (100)	43.89	28.34–65.30	0	2014	[96]
Breakfast cereals	7/54 (13.0)	0.48 ± 0.82	0.09–2.33	0	2015	[54]
Total	208/740 (28.1)			22 (3.0)		

N—Number of samples; Np—Number of positive samples; Nr—Not reported; Na—Not applicable. ^a^ Traditionally stored maize is used only as a feed. ^b^ When reported.
